# Metformin Treatment in Type 2 Diabetes in Pregnancy: An Active Controlled, Parallel-Group, Randomized, Open Label Study in Patients with Type 2 Diabetes in Pregnancy

**DOI:** 10.1155/2015/325851

**Published:** 2015-03-22

**Authors:** Jahan Ara Ainuddin, Nasim Karim, Sidra Zaheer, Syed Sanwer Ali, Anjum Ara Hasan

**Affiliations:** ^1^Department of Obstetrics and Gynecology, Dow University of Health Sciences, Karachi 74400, Pakistan; ^2^Department of Pharmacology, Medical and Dental College, Bahria University, Karachi 75500, Pakistan; ^3^School of Public Health, Dow University of Health Sciences, Karachi 75300, Pakistan; ^4^Department of Community Health Sciences, United Medical and Dental College, Karachi 74900, Pakistan; ^5^Department of Obstetrics and Gynecology, Hamdard University Hospital, Karachi 74400, Pakistan

## Abstract

*Aims.* To assess the effect of metformin and to compare it with insulin treatment in patients with type 2 diabetes in pregnancy in terms of perinatal outcome, maternal complications, additional insulin requirement, and treatment acceptability. *Methods.* In this randomized, open label study, 206 patients with type 2 diabetes in pregnancy who met the eligibility criteria were selected from the antenatal clinics. Insulin was added to metformin treatment when required, to maintain the target glycemic control. The patients were followed up till delivery. Maternal, and perinatal outcomes and pharmacotherapeutic characteristics were recorded on a proforma. *Results.* Maternal characteristics were comparable in metformin and insulin treated group. 84.9% patients in metformin group required add-on insulin therapy at mean gestational age of 26.58 ± 3.85 weeks. Less maternal weight gain (*P* < 0.001) and pregnancy induced hypertension (*P* = 0.029) were observed in metformin treated group. Small for date babies were more in metformin group (*P* < 0.01). Neonatal hypoglycemia was significantly less and so was NICU stay of >24 hours in metformin group (*P* < 0.01). Significant reduction in cost of treatment was found in metformin group. *Conclusion.* Metformin alone or with add-on insulin is an effective and cheap treatment option for patients with type 2 diabetes in pregnancy. This trial is registered with clinical trial registration number: Clinical trials.gov NCT01855763.

## 1. Introduction

Diabetes in pregnancy is becoming more common worldwide especially in South Asian countries like Pakistan. This is attributed to an increase in incidence of obesity and type 2 diabetes. Type 2 diabetes is a metabolic disorder characterized by hyperglycemia resulting from progressive deterioration in pancreatic insulin secretion, insulin resistance in tissues, and inadequate suppression of glucagon production [1]. Treatment of diabetes in pregnancy reduces serious perinatal morbidity, improves women's health related quality of life, and decreases maternal complications [2]. The increasing prevalence of type 2 diabetes in pregnancy brings with it specific challenges in terms of management. One such challenge that needs to be answered is, should metformin, an antihyperglycemic agent, be used in pregnancy with diabetes? Metformin is recommended as first-line treatment for patients with type 2 diabetes by American Diabetes Association [3]. However, metformin is not yet accepted as treatment for gestational diabetes and type 2 diabetes in pregnancy. Metformin offers a logical alternative to insulin in gestational diabetes and type 2 diabetes in pregnancy as it produces euglycemia by reducing insulin resistance, improving insulin sensitivity, reducing hepatic gluconeogenesis, and increasing peripheral glucose uptake and utilization. It also increases markers of endothelial activation which are intimately associated with insulin resistance [4–6]. Metformin, a category B drug, is not associated with fetal anomalies. Recent trial evidences support its safety and efficacy in pregnancy with gestational diabetes [7]. However as metformin crosses the placental barrier, fetal effects need to be considered [8]. Women with polycystic ovarian syndrome who continued on metformin throughout pregnancy showed decreased risk of miscarriages, preeclampsia, and gestational diabetes with better pregnancy outcomes and no increase in risk of congenital anomalies in newborns [9, 10]. There is paucity of data in terms of clinical trials relating specifically to metformin treatment in women with type 2 diabetes in pregnancy. We have a population that brings specific challenges in terms of marked insulin resistance, poor compliance to insulin treatment, and lack of financial resources. Metformin instead of conventional insulin might be an alternative, effective, and cheap treatment option for women with type 2 diabetes in pregnancy in our population. The present study was aimed at evaluating the effects of metformin therapy in type 2 diabetes in pregnancy and comparing it with standard treatment insulin.

## 2. Patients and Methods

### 2.1. Study Design

This trial was a randomized open labeled clinical phase III study (NCT01855763) with parallel assignment to patients comparing metformin with insulin treatment in type 2 diabetes in pregnancy. The study was conducted at hospitals affiliated with Dow University of Health Sciences, Karachi, Pakistan, from January 2009 till January 2014. Institutional review board and ethics committee of the university approved the study and all participants gave informed written consent.

Patients with prepregnancy diagnosed type 2 diabetes and cases of newly diagnosed overt diabetes in pregnancy (IADPSG criteria: FBS: ≥ 7.0 mmol/lit (126 mg/dL), RBS: ≥ 11.1 mmol/lit (200 mg/dL), and HbA1C ≥ 6.5%) were selected from antenatal clinics after taking informed consent. They belonged to all five major ethnic groups living in urban and rural areas of four provinces in Pakistan but now settled in urban Karachi. Women included in the study were between 20 and 48 years of age, having a singleton pregnancy continued beyond first trimester. The exclusion criteria were women who have contraindications or intolerance to metformin intake like gastrointestinal side effects and altered liver functions with or without jaundice or had hypersensitivity with the drug and women who were diagnosed as gestational diabetes or had type 1 or type 2 diabetes and were already on insulin treatment, ultrasound showing a recognized fetal anomaly, ruptured membranes in second trimester, presence of any other medical disorder, and diabetes related complications.

Randomization was done as the eligible patients enter the study with odd number assigned to metformin treatment and even number for insulin treatment irrespective of body weight and previous obstetrical history. Blinding was not possible because of different routes of administration of drugs.

### 2.2. Study Treatment

Patients were advised for dietary modifications and nutritional instructions of three meals and three snacks daily with predesigned diets according to body weight. Metformin, (Glucophage) (Merck, Pakistan) was started at dose of 500 mg/day orally and increased up to 2500 mg in three divided doses as tolerated by the patient and till glycemic control was achieved. Target blood glucose levels for glycemic control were taken as fasting blood glucose (FBS) ≤ 100 mg/dL (5.5 mmol/lit) and postprandial blood glucose levels (1.5 hours after meals) of ≤126 mg/dL (7 mmor/lit) (Diabetic Association of Pakistan). If desired, target blood glucose levels were not maintained anytime during treatment even after maximum dose of metformin; insulin was added as supplementary treatment with metformin.

Insulin (Humulin R, Humulin N (Lilly)) was prescribed as a combination of short acting and intermediate acting human insulin as twice daily injections before meals in morning (before breakfast) and in evening (before dinner) to cover for the three meals and three snacks a day or as multiple injections of short acting insulin before meals and intermediate acting insulin at bedtime depending on individual patient requirement, in order to achieve the desired glycemic targets. Dose of insulin was calculated according to body weight and gestational age. A 24-hour total insulin dose was calculated using 0.6 units/kg body weight in 1st trimester, 0.7 units/kg body weight in 2nd trimester, 0.8 units/kg body weight from 28 to 32 weeks of gestation, 0.9 units/kg body weight from 32 to 36 weeks of gestation, and 1 unit/kg body weight from 36 weeks onwards.

Patients were followed up in antenatal clinics. Iron, calcium, vitamin B12, and folic acid supplements were given to all patients. They were taught self, blood glucose monitoring using home glucose monitors and were advised to maintain a written or electronic record of blood glucose levels. Patients who could not monitor and record their blood glucose levels were tested using glucose monitors at each visit in antenatal clinic or they were admitted in day care ward for blood glucose monitoring when required. Fasting and three postprandial blood glucose levels 1.5 hours after breakfast, lunch, and dinner were recorded. Dose adjustments of drugs were made at each antenatal visit weekly or fortnightly till 36 weeks and then weekly till term or delivery. Routine obstetric care was provided at the antenatal clinics. HbAIC was done at study entry and at 36/37 weeks of pregnancy. Ultrasound was done at first visit (dating scan) then at 16–19 weeks (anomaly scan) and then monthly after 28 weeks (fetal well-being scan). Mode and time of delivery were decided at 37/38 weeks of pregnancy. Maternal and neonatal outcomes were recorded on a predesigned proforma.

## 3. Study Outcome Measures

The primary outcome measures were alive baby, neonatal hypoglycemia requiring intravenous dextrose therapy (blood glucose <25 mg/dL or <1.4 mmol/lit), and NICU admissions >24 hours.

The secondary outcome measures included birth weight, LGA/macrosomia (birth weight > 90 centile for gestational age or birth weight > 4 kg), SGA (birth weight < 10th centile for gestational age or birth weight < 2.5 kg), neonatal morbidity like transient tachypnea of newborn, respiratory distress syndrome, prematurity, sepsis, jaundice, and birth trauma, maternal glycemic control reported as mean fasting (FBS) and postprandial blood glucose levels (RBS) and HbAIC levels at 36/37 weeks of pregnancy, weight gain in pregnancy, maternal hypertensive complications including pregnancy induced hypertension (gestational hypertension) and preeclampsia (defined as B.P ≥ 140/90 on two occasions with significant proteinuria), and treatment compliance, dose, and cost of metformin and insulin treatment.

## 4. Statistical Analysis

### 4.1. Sample Size

It was estimated using the method of sample size estimation for comparing two means on www.openepi.com with mean and standard deviation of neonatal outcomes, hypoglycemia, and NICU admissions with an expected difference of 30% reduction in NICU stay and 5–10% reduction in neonatal hypoglycemia between metformin and insulin treated groups from a study done in South Asian country [11] with 5% margin of error and 95% confidence interval. We worked on 206 samples. Using PASS version 11 Chi-square for proportion with 95% CI, sample size of 206, and effect size of 0.233 with 2 degrees of freedom the power of study calculated is 0.86 from primary outcome measure, neonatal hypoglycemia, and for NICU admissions, the power of study calculated is 0.99 using effect size of 0.396.

## 5. Data Analysis

Data was analyzed using IBM SPSS version 22; the frequencies and percentages were reported for all categorical variables. Mean with SD was reported for all continuous variables. The analysis was performed to compare the metformin alone group with metformin plus insulin group and insulin alone group using two-sample independent student *t*-test and Mann-Whitney *U* test for continuous data. Chi square, Fisher Exact test, and Mann-Whitney *U* test were used for categorical data. Two-tailed tests were used for all analysis and statistical significance was considered at *P* ≤ 0.05.

## 6. Results

**Figure 1:**
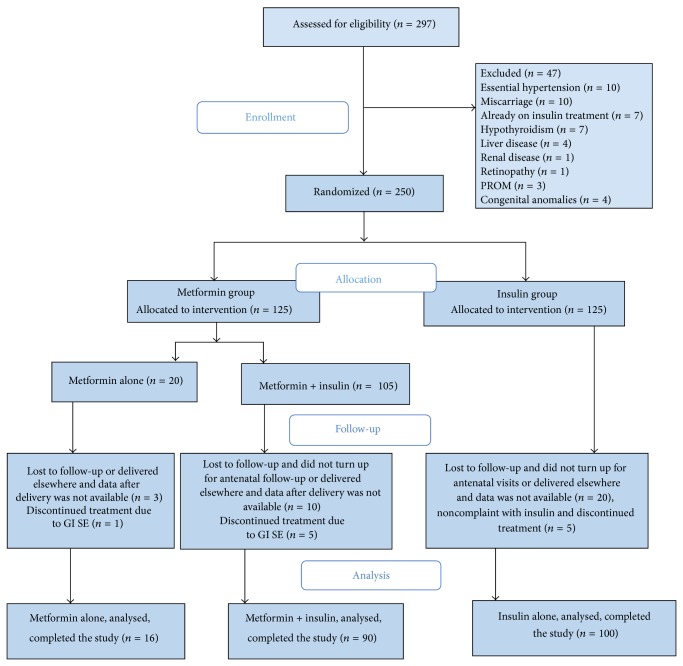
Flow diagram of study enrollment.

The overall design and subject flow through the study is illustrated in Figure 1. A total of 297 patients with type 2 diabetes in pregnancy were enrolled for the study. Of these 250 met the inclusion criteria and were randomized to treatment with metformin or insulin. 205 patients were already known cases of type 2 diabetes and were controlled on diet only or were on oral hypoglycemic agents. 45 patients were newly diagnosed cases and were classified as overt diabetes in pregnancy according to IADPSG criteria. 206 participants completed the study and their data was finally analyzed, with 106 patients in metformin group and 100 patients in insulin group (Figure 1). 84.9% patients in metformin group needed supplementary insulin treatment in varying doses to maintain glycemic control. The base line characteristics between the groups were not significantly different in metformin plus insulin and insulin alone groups; however patients in metformin alone group were younger and were of less parity (Table 1).

### 6.1. BMI and Weight Gain

BMI in early pregnancy was less in metformin alone group as compared to metformin plus insulin and insulin group (28.25 Kg/m^2^ versus 33.59 Kg/m^2^ versus 32.96 Kg/m^2^), *P* ≤0.01. Total weight gain in pregnancy was 10.38 ± 1.2 kg in metformin alone group, 10.52 ± 1.14 kg in metformin plus insulin group, and 11.80 ± 0.86 kg in insulin alone group indicating significantly less weight gain in metformin treated group, *P* ≤0.01 (Table 1).

### 6.2. Glycemic Profiles

Fasting, postprandial blood glucose levels and HbAIC levels were statistically comparable in three groups. Glycemic targets were achieved in all three groups within one week of starting treatment and maintained throughout pregnancy in the target range with no statistical difference in three groups (Table 1).

### 6.3. Maternal Outcomes

Pregnancy induced hypertension was found significantly less in metformin alone and metformin plus insulin group as compared to insulin alone group (6.2% versus 23.3% versus 36%), *P* = 0.020. However the rates of preeclampsia in three groups were 25% versus 10% versus 17%, *P* = 0.184. All women who developed preeclampsia in metformin alone group were primigravidae. Gestational age at enrolment and at delivery was not significantly different in three groups. More women delivered vaginally in metformin plus insulin group. Vaginal delivery rates were 18.8% in metformin alone, 47.8% in metformin plus insulin group, and 18% in insulin group, *P* ≤ 0.01. Cesarean section rate was 81.2% in metformin group, 52.2% in metformin plus insulin group, and 82% in insulin group showing significant reduction in cesarean section rate in metformin plus insulin group, *P* ≤ 0.01 (Table 2).

### 6.4. Neonatal Outcomes

There were no perinatal deaths and all babies were born alive in three groups. Mean birth weights was statistically the same in all groups. Significantly more small for gestational age babies were found in metformin treated groups as compared to insulin alone group (31.2% versus 14.4% versus 2%), *P* ≤ 0.01. Incidence of macrosomia was statistically the same in three groups (12.5% versus 33.3% versus 27%), *P* = 0.208. Transient tachypnea of newborn was found in 12.5% versus 6.66% versus 18% in metformin alone, metformin plus insulin, and insulin alone group, *P* = 0.063. Comparable rates of RDS were found in three groups with no statistical difference but lower rates in metformin plus insulin group (12.5% versus 5.6% versus 11%), *P* = 0.355. 10% neonates in insulin alone group developed neonatal sepsis as compared to 3.3% in metformin plus insulin group, *P* = 0.094. Two newborns in metformin alone group and 2 in insulin group had birth trauma with clavicle fracture in two neonates and cephalhematoma associated with vacuum delivery in other 2 neonates; all of them had birth weight of > 4 kg. NICU stay was significantly less in metformin alone and metformin plus insulin group as compared to insulin alone group, *P* ≤ 0.01 (Table 3).

Significantly less neonatal jaundice was observed in metformin alone and metformin plus insulin group as compared to insulin group, *P* = 0.021. Apgar scores at 5 minutes of birth were statistically comparable in 3 groups. Significantly less neonatal hypoglycemia was found in metformin plus insulin group, *P* ≤ 0.01, and mean blood glucose levels at birth were also better in metformin plus insulin group (Table 3).

### 6.5. Pharmacotherapeutic Characteristics

Treatment compliance was good with oral metformin treatment than with insulin injections (see Supplementary Table in Supplementary Material available online at http://dx.doi.org/10.1155/2015/325851). Mean dose of metformin throughout pregnancy was 2.21 ± 0.63 in metformin alone group and 2.291 ± 0.63 grams per day in metformin plus insulin group, *P* = 0.665. 84.9% patients required additional insulin in metformin group to maintain glycemic targets in mean dose of 23.64 ± 4.61 units per day. Mean dose of insulin required in insulin alone group was 77.62 ± 12.69 units per day which was significantly higher than that required in metformin group as add-on therapy, *P* ≤ 0.01. Mean gestational age at which insulin was add-on in metformin group was 26.58 ± 3.8 weeks of pregnancy while insulin was started at mean gestational age of 9.55 ± 5.2 weeks in insulin group, *P* ≤ 0.01. Cost of treatment was significantly less in metformin alone and metformin plus insulin group as compared to insulin alone group, *P* ≤ 0.01 (Table 4).

### 6.6. Maternal and Neonatal Outcomes Adjusted for BMI

Out of 206 patients 50 had BMI < 30 and 156 had BMI > 30 indicating association of obesity with type 2 diabetes. Total weight gain in pregnancy was less in metformin alone group with BMI > 30, *P* ≤ 0.01. Mean dose of add-on insulin was 27.63 ± 6.03 units in women with BMI < 30 and 22.84 ± 3.78 in women with BMI > 30, *P* = 0.519, indicating no statistical difference in insulin dose, in groups stratified according to BMI. However mean gestational age at which insulin started was earlier in women with BMI > 30 as compared to women with BMI < 30, *P* ≤ 0.01. Similarly pregnancy induced hypertension was less in women with BMI < 30 as compared to women with BMI > 30, *P* = 0.011. There were no statistical differences in NICU stay and neonatal hypoglycemia in two groups stratified according to BMI, *P* = 0.249 and *P* = 0.699, respectively (Table 5).

## 7. Discussion

This randomized clinical study reports the results of metformin versus insulin treatment in women with type 2 diabetes in pregnancy. The study was conducted in a population setting with high prevalence of diabetes to meet the needs in a background of low economic resources where health facilities especially medicines are not always available free of cost. Patients with type 2 diabetes in pregnancy were selected with strict inclusion criteria; that is, they were not on insulin treatment and did not have complications associated with diabetes. Although duration of diabetes was not taken into account, the study patients, either had newly diagnosed overt diabetes detected in this pregnancy or were known cases of type 2 diabetes, who were on diet control or on oral antihyperglycemic agents, with duration of diabetes not more than 5 years.

In mean age and BMI the difference was statistically insignificant in metformin and insulin treated groups but patients in metformin alone group were younger and most had BMI < 30. Older age and multiparity with BMI > 30 were also observed in previous studies as in our study [12–14]. Total weight gain in pregnancy was significantly less in metformin treated patients than in insulin treated patients. Similar results were reported by Rowan et al. [7, 15–17]. This effect of less weight gain was observed more in women with BMI > 30 in our study. Glycemic targets were achieved within one week of starting treatment and maintained throughout pregnancy. Similar findings were reported by Rowan et al., Hickman, Tertti, and others [7, 11, 18–20] indicating metformin to be effective in achieving diabetes control.

We found significantly less pregnancy induced hypertension in metformin group as compared to insulin group, these findings being related to the effect of metformin on reducing endothelial activation and maternal inflammatory response to insulin resistance [21]. However more preeclampsia was found in elderly obese primigravidae in metformin alone group. No differences in rates of maternal hypertensive complications were found in other studies [7, 14, 16, 22, 23]. However, higher incidence of preeclampsia was reported by Hellmuth et al. in metformin treated patients [24]. Patients with BMI > 30 were found to have more pregnancy induced hypertension than patients with BMI < 30. Metformin treatment however is associated with significantly less occurrence of pregnancy induced hypertension in women with BMI > 30 in our study.

Patients were enrolled for study at around 10 weeks of gestation with a viable pregnancy. Mean gestational age at delivery was nearly the same around 37 weeks. More women delivered vaginally in metformin plus insulin group as compared to insulin alone group (47.77% versus 18%) and similarly cesarean section rate was statistically lower in metformin treated group. No differences in cesarean section and vaginal delivery rates were reported in other studies [7, 13, 15, 24]. However, 81.25% patients required cesarean section in metformin alone group mainly because of association with preeclampsia and intrauterine growth retardation.

No perinatal deaths occurred partly because congenital anomalies were excluded from the study and tight glycemic control was maintained, besides keeping a low threshold for cesarean section in cases of presumed fetal compromise. There was no difference in neonatal outcomes like birth weight, Apgar scores, large for gestational age infants, and respiratory distress syndrome in three groups. However significantly more small for gestational age infants were found in metformin group as compared to insulin group; association of preterm delivery before 37 weeks and birth weight < 10th centile for gestational age were contributing factors in metformin group. Rowan et al. reported more spontaneous preterm births in metformin group than in insulin treated group but same number of small for date infants in both groups in MIG trial [7]. Neonatal complications like transient tachypnea of newborn, neonatal sepsis, neonatal intensive care unit stay, and neonatal hypoglycemia were found significantly less in metformin treated group when compared with insulin group. Similar results were reported in other studies [7, 12, 13, 15, 18, 19, 23]. Women with BMI > 30 had more neonatal complications in insulin treated group as compared to metformin treated group indicating protective effect of metformin in reducing neonatal hypoglycemia and thus NICU admissions.

Patients achieved adequate glycemic control with mean dose of 2.5 grams of metformin, and few required dose limitation due to gastrointestinal side effects. These were those who required early add-on insulin therapy. Mean gestational age at which insulin started in metformin treated group was 26 weeks in our study. 84.9% patients in metformin group required add-on insulin therapy in our study. Patients with BMI > 30 required add-on insulin earlier than those with BMI < 30 in metformin treated group. BMI appeared to be a predictor for need of add-on insulin in metformin treated group, as patients who were controlled on metformin alone had mean BMI of less than 30. Insulin was add-on with metformin to maintain glycemic control when insulin resistance increases in pregnancy that is around 26–28 weeks of pregnancy. In metformin treated group, patients with early pregnancy BMI of < 30 did not require add-on insulin or required add-on insulin at a later gestational age in our study. Ijas et al. reported similar results in their study [23]. Other studies in gestational diabetes show varying figures in terms of supplemental insulin requirement. Rowan et al. reported 46.3%, Viollet et al. 18%, and Moore et al. 34.7% [7, 21, 25]. Coetzee and Jackson reported that 28.6% patients with type 2 diabetes in pregnancy required supplemental insulin with metformin [26]. Mohammed et al. in a retrospective analysis, however, reported that 95% patients with type 2 diabetes in pregnancy required additional insulin to maintain glycemic control [14]. These disparities in need for add-on insulin with metformin may be explained by differences in populations studied because diabetes and its control vary widely in different populations related to their genetic and phenotypic makeup. WHO ranks Pakistan as a high prevalent area for diabetes with patients exhibiting high insulin resistance and obesity [27]. This explains the reason of failure of metformin alone to achieve glycemic control in these patients. Mean dose of add-on insulin was significantly less in metformin group as compared to insulin group and so the financial benefit achieved was significant in metformin group. This financial benefit is also shown in gestational diabetes part of this study [28].

## 8. Conclusion

Metformin is an effective treatment option for women with type 2 diabetes in pregnancy with or without add-on insulin who require pharmacological treatment for glycemic control in our resource poor setting.

Metformin has advantages over insulin such as less maternal weight gain, no maternal hypoglycemia, being cheap, being oral therapy, and requiring no vigorous monitoring and frequent hospital admissions with good compliance and acceptability. Metformin treatment when compared with insulin treatment showed less maternal hypertensive complications and less risk of neonatal hypoglycemia with few neonatal intensive care admissions. Metformin treatment is suitable for nonobese type 2 diabetes patients in pregnancy without complications. Metformin treatment in type 2 diabetes in pregnancy required lower dose of add-on insulin, at a later gestational age for maintaining glycemic control when compared with insulin treatment.

Study limitations included duration of diabetes that was not taken into account in the present study; besides congenital anomalies were excluded from the study and longer infant follow-up was not carried out.

The findings of the trial are important in recommending metformin for treatment of type 2 diabetes in pregnancy in a selected population.

## Supplementary Material

Patients in metformin group had good treatment compliance when compared to insulin group P<0.01. Only six patients had gastro intestinal side effects resulting in dose limitation in metformin group. 92.4% patients opted for metformin treatment in next pregnancy while all patients in metformin group considered metformin treatment as easy and cheap. Doing finger pricks for blood glucose monitoring was considered as the most difficult part of treatment in both groups.

## Figures and Tables

**Table 1:** Baseline demographic and maternal characteristics in treatment groups (*n* = 206).

Parameters	Group	Mean ± SD	*P* value^a^	*P* value^b^	*P* value^c^
Age (years)	Metformin alone	31.75 ± 2.82	0.05^*^		
	Metformin + insulin	34.09 ± 3.51		0.07	
	Insulin alone	33.73 ± 2.95			0.956

Parity	Metformin alone	2.56 ± 1.15	0.550		
	Metformin + insulin	2.83 ± 1.18		0.038^*^	
	Insulin alone	3.18 ± 1.15			0.025^*^

BMI-early pregnancy (kg/m^2^)	Metformin alone	28.25 ± 1.98	<0.01^*^		
	Metformin + insulin	33.59 ± 3.97		<0.01^*^	
	Insulin alone	32.96 ± 4.04			0.171

BMI-late pregnancy (kg/m^2^)	Metformin alone	32.47 ± 2.19	<0.01^*^		
	Metformin + insulin	38.09 ± 4.26		<0.01^*^	
	Insulin alone	38.01 ± 4.18			0.714

Total weight gain in pregnancy (Kg)	Metformin alone	10.38 ± 1.20	0.868		
	Metformin + insulin	10.52 ± 1.14		<0.01^*^	
	Insulin alone	11.80 ± 0.86			<0.01^*^

FBS at starting treatment (mg/dl)	Metformin alone	138.06 ± 45.58	0.304		
	Metformin + insulin	144.14 ± 29.64		0.877	
	Insulin alone	139.85 ± 29.43			0.155

RBS at starting treatment (mg/dl)	Metformin alone	192.88 ± 25.56	0.326		
	Metformin + insulin	195.03 ± 21.69		0.041^*^	
	Insulin alone	201.21 ± 16.78			0.158

Mean FBS throughout pregnancy (mg/dl)	Metformin alone	97.87 ± 3.83	0.586		
	Metformin + insulin	97.50 ± 3.35		0.672	
	Insulin alone	97.55 ± 3.29			0.962

Mean RBS throughout pregnancy (mg/dl)	Metformin alone	136.79 ± 5.34	0.176		
	Metformin + insulin	134.79 ± 5.69		0.237	
	Insulin alone	135.31 ± 4.64			0.944

*P* value calculated by using independent *t*-test∖Mann-Whitney *U* test.

^a^Comparison between means of metformin alone and metformin + insulin groups.

^b^Comparison between means of metformin alone and insulin alone.

^c^Comparison between means of metformin + insulin and insulin alone.

^*^Significant level ≤0.05.

FBS: fasting blood glucose.

RBS: random blood glucose.

**Table 2:** Maternal outcomes in treatment groups (*n* = 206).

Parameters	Metformin alone	Metformin + insulin	Insulin alone	*P* value
	*n* = 16	*n* = 90	*n* = 100	
	*n* (%)	*n* (%)	*n* (%)	
Maternal hypertensive complications in pregnancy				
Pregnancy induced hypertension				
Yes	1 (6.2)	21 (23.3)	36 (36.0)	0.020^*^
No	15 (93.8)	69 (76.7)	64 (64.0)	
Preeclampsia				
Yes	4 (25.0)	9 (10.0)	17 (17.0)	0.184
No	12 (75.0)	81 (90.0)	83 (83.0)	

Labor outcomes				
Normal vaginal delivery				
Yes	3 (18.8)	43 (47.8)	18 (18.0)	<0.01^*^
No	13 (81.2)	47 (52.2)	82 (82.0)	
LSCS				
Yes	13 (81.2)	47 (52.2)	82 (82.0)	<0.01^*^
No	3 (18.8)	43 (47.8)	18 (18.0)	

Obstetric outcomes				
	Mean ± SD	*P* value^a^	*P* value^b^	*P* value^c^

Gestational age at enrolment (weeks)				
Metformin alone	10.75 ± 5.98	0.876		
Metformin + insulin	10.09 ± 4.86		0.601	
Insulin alone	9.57 ± 5.20			0.661
Gestational age at delivery (weeks)				
Metformin alone	36.19 ± 1.68	0.409		
Metformin + insulin	36.86 ± 1.35		0.134	
Insulin alone	37.06 ± 1.22			0.465

*P* value calculated by using Chi-square test∖Mann-Whitney *U* test.

^a^Comparison between means of metformin alone and metformin + insulin groups.

^b^Comparison between means of metformin alone and insulin alone groups.

^c^Comparison between means of metformin + insulin and insulin alone groups.

^*^Significant level ≤0.05.

**Table 3:** Neonatal outcomes in treatment groups (*n* = 206).

Parameters	Metformin alone	Metformin + insulin	Insulin alone	*P* value
	*n* = 16	*n* = 90	*n* = 100	
	*n* (%)	*n* (%)	*n* (%)	
Born alive				
Yes	16 (100)	90 (100)	100 (100)	N/A
No	—	—	—	
Small for gestational				
Yes	5 (31.2)	13 (14.4)	2 (2.0)	<0.01^*^
No	11 (68.8)	77 (85.6)	98 (98.0)	
Large for gestational				
Yes	2 (12.5)	30 (33.3)	27 (27.0)	0.208
No	14 (87.5)	60 (66.7)	73 (73.0)	
Birth trauma				
Yes	2 (12.5)	0 (0)	2 (2.0)	0.016^*^
No	14 (87.5)	90 (100)	98 (98.0)	
NICU stay				
Yes	7 (43.8)	21 (23.3)	69 (69.0)	<0.01^*^
No	9 (56.2)	69 (76.7)	31 (31.0)	
Neonatal jaundice				
Yes	2 (12.5)	9 (10.0)	25 (25.0)	0.021^*^
No	14 (87.5)	81 (90.0)	75 (75.0)	
TTN				
Yes	2 (12.5)	6 (6.7)	18 (18.0)	0.063
No	14 (87.5)	84 (93.3)	82 (82.0)	
RDS				
Yes	2 (12.5)	5 (5.6)	11 (11.0)	0.355
No	14 (87.5)	85 (94.4)	89 (89.0)	
Neonatal sepsis				
Yes	0 (0)	3 (3.3)	10 (10.0)	0.094
No	16 (100)	87 (96.7)	90 (90.0)	
Neonatal hypoglycemia				
Yes	4 (25.0)	7 (7.8)	30 (30.0)	<0.01^*^
No	12 (75.0)	83 (92.2)	70 (70.0)	

Continuous measure outcomes				
	Mean ± SD	*P* value^a^	*P* value^b^	*P* value^c^

Birth weight (kg)				
Metformin alone	3.03 ± 0.65	0.305		
Metformin + insulin	3.34 ± 0.68		0.345	
Insulin alone	3.41 ± 0.56			0.985
Mean blood glucose level at birth (mg/dl)				
Metformin alone	39.25 ± 9.32	0.062		
Metformin + insulin	45.41 ± 7.94		0.335	
Insulin alone	40.91 ± 14.81			0.282
Apgar score at 5 minutes				
Metformin alone	8.31 ± 1.01	0.929		
Metformin + insulin	8.27 ± 0.94		0.228	
Insulin alone	8.05 ± 0.90			0.048^*^

*P* value calculated by using Chi-square∖Fisher Exact∖Mann-Whitney *U* test.

^a^Comparison between means of metformin alone and metformin + insulin groups.

^b^Comparison between means of metformin alone and insulin alone groups.

^c^Comparison between means of metformin + insulin and insulin alone groups.

^*^Significant level ≤0.05.

TTN: transient tachypnea of newborn.

RDS: respiratory distress syndrome.

NICU: neonatal intensive care unit.

**Table 4:** Pharmacotherapeutic characteristics in treatment groups (*n* = 206).

Parameters	Group	Mean ± SD	*P* value^a^	*P* value^b^	*P* value^c^
Dose of drug					
Metformin: mean dose grams/day	Metformin alone	2.21 ± 0.63	0.665		
	Metformin + insulin	2.29 ± 0.64		—	
	Insulin alone	—			—
Insulin: mean dose units/day	Metformin alone	—	—		
	Metformin + insulin	23.69 ± 4.61		—	
	Insulin alone	77.62 ± 12.69			<0.01^*^
Mean gestational age when insulin started (weeks)	Metformin alone	—	—		
	Metformin + insulin	26.58 ± 3.85		—	
	Insulin alone	9.55 ± 5.21			<0.01^*^

Financial benefits					
Total cost throughout pregnancy (PKR)	Metformin alone	448 ± 131	<0.01^*^		
	Metformin + insulin	5004 ± 754		<0.01^*^	
	Insulin alone	10976 ± 6339			<0.01^*^
Total cost throughout pregnancy (USD)	Metformin alone	4.39 ± 1.01	<0.01^*^		
	Metformin + insulin	49.05 ± 7.39		<0.01^*^	
	Insulin alone	107.60 ± 62.14			<0.01^*^

*P* value calculated by using Mann-Whitney *U* test.

^a^Comparison between means of metformin alone and metformin + insulin groups.

^b^Comparison between means of metformin alone and insulin alone groups.

^c^Comparison between means of metformin + insulin and insulin alone groups.

^*^Significant level ≤0.05.

PKR: Pakistani rupee.

USD: US dollar.

**Table 5:** Maternal and neonatal characteristics in treatment groups adjusted for BMI (*n* = 206).

Parameters	Normal (BMI ≤ 30), *n* = 50				Obese (BMI > 30), *n* = 156				*P* value^d^
Group	Mean ± SD	*P* value^a^	*P* value^b^	*P* value^c^	Mean ± SD	*P* value^a^	*P* value^b^	*P* value^c^	
Total weight gain in pregnancy (Kg)									<0.01^*^
Metformin alone	10.53 ± 0.87	0.031^*^			9.66 ± 2.30	0.664			
Metformin + insulin	9.12 ± 1.31		<0.01^*^		10.82 ± 0.84		0.011^*^		
Insulin alone	11.71 ± 0.95			<0.01^*^	11.82 ± 0.84			<0.01^*^	

Pharmacotherapeutic characteristics									
Insulin: mean dose units/day									0.519
Metformin alone	—	—			—	—			
Metformin + insulin	27.63 ± 6.03		—		22.84 ± 3.78		—		
Insulin alone	75.52 ± 14.93			<0.01^*^	78.18 ± 12.06			<0.01^*^	
Mean gestational age when insulin started (weeks)									<0.01^*^
Metformin alone	—	—			—	—			
Metformin + insulin	28.31 ± 1.95		—		26.20 ± 4.06		—		
Insulin alone	8.48 ± 2.50			<0.01^*^	9.84 ± 5.69			<0.01^*^	

	Metformin alone	Metformin + insulin	Insulin alone	*P* value	Metformin alone	Metformin + insulin	Insulin alone	*P* value	
	(*n* = 13)	(*n* = 16)	(*n* = 21)		(*n* = 3)	(*n* = 74)	(*n* = 79)		

Maternal characteristics									
Pregnancy Induced Hypertension									0.011^*^
Yes	1 (7.7)	1 (6.2)	5 (23.8)	0.238	0 (0)	20 (27.0)	31 (39.2)	0.035^*^	
No	12 (92.3)	15 (93.8)	16 (76.2)		3 (100)	54 (73.0)	48 (60.8)		

Neonatal characteristics									
NICU stay									0.249
Yes	7 (53.8)	2 (12.5)	11 (52.4)	0.029^*^	0 (0)	19 (25.7)	58 (73.4)	<0.01^*^	
No	6 (46.2)	14 (87.5)	10 (47.6)		3 (100)	55 (74.3)	21 (26.6)		
Neonatal hypoglycemia									0.699
Yes	4 (30.8)	0 (0)	5 (23.8)	0.066	0 (0)	7 (9.5)	25 (31.6)	<0.01^*^	
No	9 (69.2)	16 (100)	16 (76.2)		3 (100)	67 (90.5)	54 (68.4)		

*P* value calculated by using Mann-Whitney *U* test∖Chi-square∖Fisher Exact test.

^a^Comparison between means of metformin alone and metformin + insulin groups.

^b^Comparison between means of metformin alone and insulin alone groups.

^c^Comparison between means of metformin + insulin and insulin alone groups.

^d^Comparison between BMI with parameters only.

^*^Significant level ≤0.05.
