# Supplementary material for: Metformin Treatment in Type 2 Diabetes in Pregnancy: An Active Controlled, Parallel-Group, Randomized, Open Label Study in Patients with Type 2 Diabetes in Pregnancy
Source: J Diabetes Res. 2015 Mar 22;2015:325851. doi: 10.1155/2015/325851 (PMC4385634; doi:10.1155/2015/325851)
Supplement: Supplementary file 1 — Patients in metformin group had good treatment compliance when compared to insulin group P<0.01. Only six patients had gastro intestinal side effects resulting in dose limitation in metformin group. 92.4% patients opted for metformin treatment in next pregnancy while all patients in metformin group considered metformin treatment as easy and cheap. Doing finger pricks for blood glucose monitoring was considered as the most difficult part of treatment in both groups. [file 325851.f1.docx]

**TREATMENT COMPLIANCE**

| **Treatment compliance and side effects** | **Metformin group**  **n=106** | **Insulin group**  **n=100** | **p-value^2,3,4^** |
| --- | --- | --- | --- |
|  | **N (%)** | **N (%)** |  |
| **How often did you forget to take treatment?** |  |  |  |
| Never or rarely | 87 (82.1) | 60 (60) | <0.01 |
| 2 to 4 times/week | 19 (17.9) | 40 (40) | <0.01 |
| **Side effects** |  |  |  |
| GI side effect resulting in dose limitation | 6 (5.6) | 0 (0) | 0.2 |
| GI side effect resulting in treatment cessation | 0 (0) | 0 (0) | - |
| Lactic acidosis | 0 (0) | 0 (0) | - |

| **Treatment acceptability** | **Metformin group**  **n=106** | **Insulin group**  **n=100** | **p-value^2,3,4^** |
| --- | --- | --- | --- |
|  | **N (%)** | **N (%)** |  |
| **Which medication would you choose in next pregnancy?** |  |  |  |
| Metformin | 98(92.4) | 12 (12) | <0.01 |
| Insulin | 4 (3.8) | 53(53) | <0.01 |
| not sure | 4 (3.8) | 35 (35) | <0.01 |
| **Which part of diabetes treatment was easy?** |  |  |  |
| Doing finger pricks | 0 (0) | 0 (0) | N.A |
| Diet control | 0 (0) | 25 (25) | <0.01 |
| Drug treatment | 106 (100) | 75 (75) | <0.01 |
| **Which part of diabetes treatment was difficult?** |  |  |  |
| Doing finger pricks | 88 (83.1) | 24 (24) | <0.01 |
| Diet control | 8 (7.5) | 10 (10) | 0.004 |
| Drug treatment | 10 (9.4) | 66 (66) | <0.01 |

^1^ Mean ± Standard Deviation Reported for all Continuous variables

^2^p- value was calculate using two independent sample t-test

^3^p-value was calculated using chi-square test of association

^4^ p <0.05 considered as significant
